# Hsa_circ_0003748 promotes disease progression in rheumatic valvular heart disease by sponging miR‐577

**DOI:** 10.1002/jcla.24487

**Published:** 2022-05-09

**Authors:** Xiaoyun Zhang, Yakun Gao, Hongyu Wu, Yong Mao

**Affiliations:** ^1^ Cardio‐vascular Surgery Ningbo First Hospital Ningbo China

**Keywords:** biological function, biomarker, has_circ_0003748, miR‐577, rheumatic valvular heart disease

## Abstract

The diagnosis and treatment of rheumatic valvular heart disease (RVHD) require substantial improvements. Studies found that circular RNAs (circRNAs) are involved in the progression of cardiovascular diseases. We screened target hsa_circ_0003748 by circRNA microarrays uploaded to a database. We used fluorescence in situ hybridization to determine the cellular location of hsa_circ_0003748. A dual‐luciferase reporter gene assay revealed that has_circ_0003748 might bind the miRNA miR‐577. In hVIC cells (an RVHD cell line), Cell Counting Kit‐8, Transwell, and flow cytometry assays measured proliferation, migration, and cell cycle and apoptosis, respectively. We found that hsa_circ_0003748 was localized in the cytoplasm; hsa_circ_0003748 promoted the proliferation and migration of hVIC cells, arrested the cell cycle in the G2/M phase, and inhibited apoptosis. These phenomena may result from hsa_circ_0003748 promoting RVHD after sponging miR‐577. Bioinformatic analysis revealed that hsa_circ_0003748 might affect RVHD progression by affecting transcription and the MAPK signaling pathway, the Ras signaling pathway, the cAMP signaling pathway, the Rap1 signaling pathway, and other signaling pathways.

## Graphical abstract

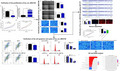

## 1 INTRODUCTION

**Background:** Rheumatic valvular heart disease (RVHD) is caused by rheumatic fever that might affect the heart valves or cause pericarditis, including the pericardium, myocardium, and endocardium.^1^, ^2^ Severe cases are often characterized by non‐purulent inflammation, heart failure, pulmonary edema, and hemoptysis. The treatment is challenging, and the outcomes are poor.^3^, ^4^ If rheumatic fever occurs repeatedly, it often causes severe damage to the endocardium, especially the endocardial tissue of the mitral valve, mechanical damage caused by turbulent flow, and platelet accumulation.^5^, ^6^, ^7^ RVHD should be diagnosed early, and targeted treatment should be carried out as soon as possible to improve outcomes.

Circular RNAs (circRNAs) differ from linear RNA in that the 5’ and 3’ ends are linked end‐to‐end to form a covalently closed single‐stranded circular molecule.^8^ CircRNAs are usually composed of exons, introns, or both and are widely present in several organisms. They are evolutionarily conserved, relatively stable, not easily degraded by exonuclease, and have tissue‐specific and developmental stage specificity.^9^, ^10^, ^11^ The biological functions of circRNAs include microRNA sponging, single circRNA regulatory protein binding, circRNA−protein interactions, and coding.^9^, ^12^, ^13^ CircRNAs participate in normal physiological activities and many diseases. Several studies found that circRNAs are associated with the progression of cardiovascular diseases, tumors, diabetes, and nervous system diseases.^14^, ^15^, ^16^, ^17^

There are few studies on circRNA in RVHD; therefore, studies of the effect of circRNA on RVHD are urgently needed to improve the diagnosis and treatment. The present study identified the target circRNA hsa_circ_0003748 from the Gene Expression Omnibus (GEO) database (GSE168932, https://www.ncbi.nlm.nih.gov/geo/query/acc.cgi?acc=GSE168932) and performed in vitro experiments to explore the effect of has_circ_0003748 on the occurrence and development of RVHD after hsa_circ_0003748 sponging miR‐577. The possible functions and signaling pathways involved in the RVHD process were analyzed using Gene Ontology (GO) and Kyoto Encyclopedia of Genes and Genomes (KEGG) after hsa_circ_0003748 sponges miR‐577.

## 2 MATERIALS AND METHODS

### 2.1 Data mining

We screened circRNA microarray data that had been uploaded to the GEO database (GSE168932, https://www.ncbi.nlm.nih.gov/geo/query/acc.cgi?acc=GSE168932). Candidate circRNAs with fold‐change >2 and *p* < 0.05 were selected. We performed a literature search of circRNAs that had not been studied to design specific primers and verified these primers using small samples. Finally, we selected hsa_circ_0003748 as the research object (fold‐change = 2.774, *p* = 0.01).

### 2.2 Cell culture and transfection

The hVIC cell line is an immortalized primary line constructed from the heart valve interstitium of RVHD patients (iCell, China). We cultured hVICs in primary mesenchymal cell basal medium (iCell) containing 1% primary mesenchymal cell culture supplement (iCell), 1% penicillin/streptomycin (Life Technologies, USA), and 10% fetal bovine serum (Gibco, USA). Cells were grown at 37°C in an incubator (Thermo Fisher, USA) containing 5% CO_2_. When cells were in a logarithmic growth phase, we transfected has_circ_0001204 (siRNA) and siRNA negative control (NC) (GenePharma, China). We also transfected empty pGL3 vector and empty pcDNA3.1 vector overexpressing hsa_circ_0003748 recombinant plasmids (Geneseed Biotech Co., Ltd., China). According to the manufacturer's protocol, cells were transfected using Lipofectamine 3000 (Invitrogen, USA).

### 2.3 Real‐time quantitative polymerase chain reaction

Relative expression levels of hsa_circ_0003748 were measured using GoTaq qPCR Master Mix (Promega, USA) reagent, and RT‐qPCR detection was performed on Applied Biosystems 7500 Real‐time PCR system (ThermoFisher Scientific, USA). To determine relative expression levels, we used glyceraldehyde‐3‐phosphate dehydrogenase (GAPDH) mRNA as an external reference. The forward primer for RT‐qPCR detection of hsa_circ_0003748 was 5'‐GTGGCATGCAGCCATAAGTT‐3', and the reverse primer was 5'‐GTACTGGTTCCGATGCTTTGC‐3'; the forward primer for RT‐qPCR detection of GAPDH was 5'‐GCACCGTCAAGGCTGAGAAC‐3', and the reverse primer was 5'‐TGGTGAAGACGCCAGTGGA‐3'. The calculated value of 2^−ΔCq^ reflects the relative expression level of hsa_circ_0003748. The larger the 2^−ΔCq^, the higher the relative expression level.

### 2.4 Cell counting kit 8 assay

We first trypsinized the transfected cells and seeded 5000 cells per 96‐well plate (Corning, USA). Cell viability was measured separately at times 1~5 days after seeding. We added 10 μl of cell counting kit 8 (CCK‐8) reagent to each well according to the manufacturer's instructions (Dojindo, Japan) and incubated them for 4 h in an incubator protected from light. Absorbance at 450 nm was then measured using a SpectraMax M5 Microplate Reader (Molecular Devices, USA).

### 2.5 Wound‐healing assay

After transfection, the cells were cultured to confluence, and then an artificial wound was created by scraping the cells with a 200‐μl pipette tip. Floating cells were washed off with phosphate‐buffered saline (PBS), and pictures of scratches were taken at 0 and 48 hours. The ImagePro Plus v6.0 software package (Media Cybernetics Inc., USA) measured the cell migration distance and calculated the cell migration rate.

### 2.6 Transwell assay

Transfected cells were digested with trypsin, and the digested cells were resuspended and seeded into the upper chamber of the Transwell well (Costar, USA) at 8 × 10^4^ cells per well. We placed the upper chamber into 24‐well plates containing 20% fetal bovine serum‐containing complete medium. Cells were fixed with 4% paraformaldehyde for 30 min after 24 h incubation in the incubator. Cells were then stained with 0.1% crystal violet solution for 30 min. Finally, we selected a suitable field of view under an inverted microscope to count the cells.

### 2.7 Cell apoptosis and cell cycle assays

Transfected cells were trypsinized without EDTA and resuspended in binding buffer for apoptosis assays. The cells were then stained with an Annexin V‐FITC/PI Apoptosis Kit (Multi Sciences, China) and incubated in the dark for 15 minutes at room temperature. Apoptosis was measured using a FACSCalibur flow cytometer (Becton Dickinson Co., USA).

For cell cycle assays, hVICs were starved in a serum‐free medium for cell cycle synchronization before transfection, harvested, washed in PBS, then fixed with 70% ethanol, and stored in a −20°C freezer overnight. The cells were then washed with pre‐cooled PBS and incubated with 1 mL of PI/RNase staining buffer (Multi Sciences, China) for 30 min in the dark. Finally, cell cycle distribution was measured using a FACSCalibur flow cytometer (Becton Dickinson Co., USA).

### 2.8 Fluorescence in situ hybridization assay

A specific probe for hsa_circ_0003748 was designed (RiboBio, China), the nucleus was stained with blue dye, and the cytoplasm was stained with red dye. Following the manufacturer's instructions (RiboBio, China), we observed fluorescent staining under a fluorescence microscope (Leica, Germany).

### 2.9 Dual‐luciferase reporter assay

Before transfection, cells were plated at 1 × 10^4^ cells/well in 24‐well plates. After the hsa_circ_0003748 wild‐type and mutant reporter vectors targeting miR‐577 were constructed, the changes in luciferase activity were calculated as the activity values of firefly and renilla luciferases. All operations were performed according to the manufacturer's instructions (GenePharma, China).

### 2.10 GO and KEGG bioinformatics analysis

We analyzed the related biological functions and signaling pathways affected by hsa_circ_0003748 sponging miR‐577 by GO and KEGG, which were completed by DIANA‐miRPath online software (http://diana.imis.athena‐innovation.gr/DianaTools/ index.php).

### 2.11 Statistical analysis

Statistical analysis was performed by the Statistical Program for the Social Sciences (SPSS) 20.0 (SPSS, USA) and GraphPad Prism 6.0 (GraphPad Software, USA) software. Appropriate statistical methods were selected to process the data, and each statistical result was evaluated as statistically significant when the *p* value was less than 0.05.

## 3 Results

### 3.1 Hsa_circ_0003748 promotes RVHD progression

**FIGURE 1:**
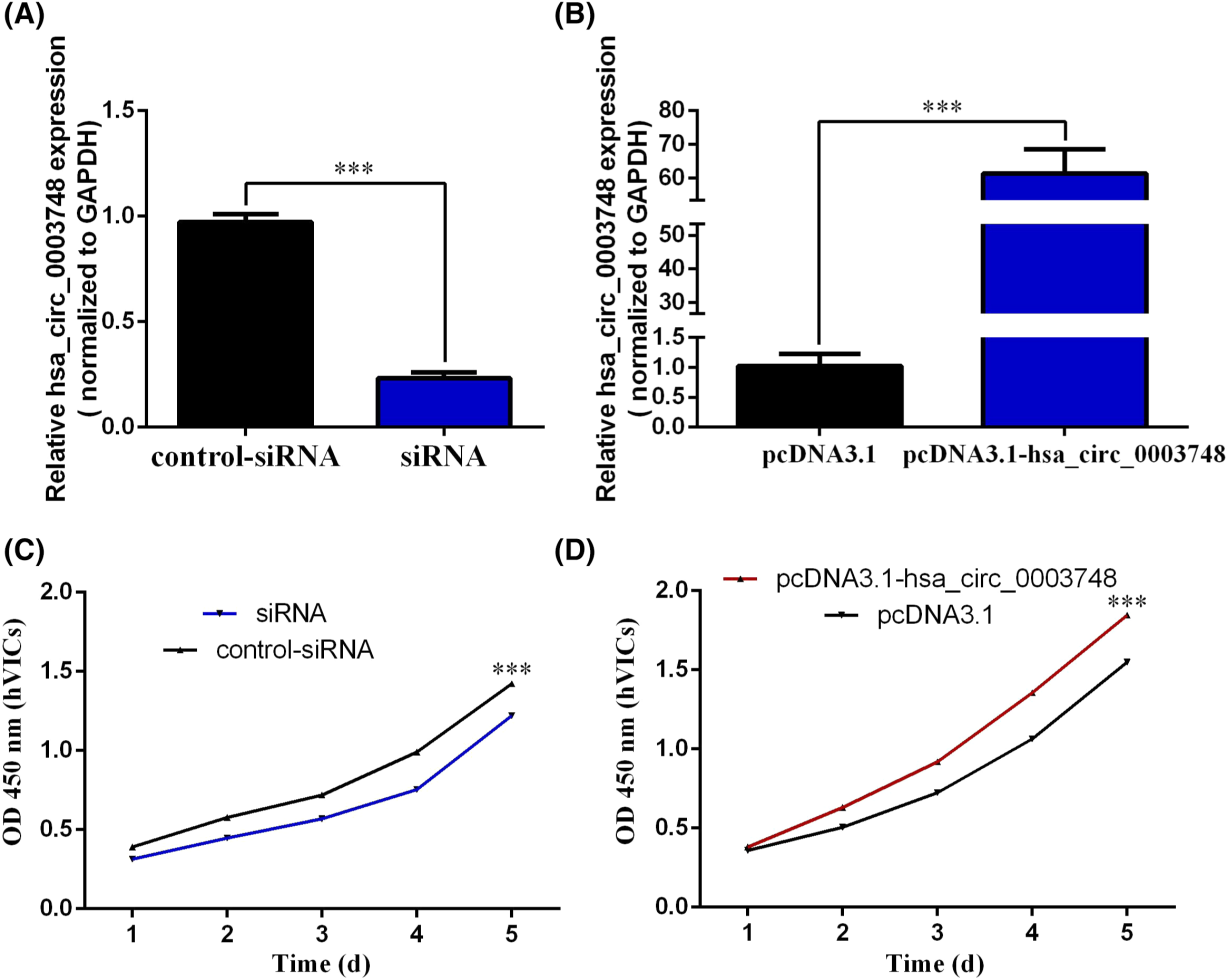
Hsa_circ_0003748 promotes hVICs cell proliferation. (A) Expression levels after silencing hsa_circ_0003748. (B) Expression levels after overexpressing hsa_circ_0003748. (C) Growth curve of hVICs cells after silencing hsa_circ_0003748. (D) Growth curve of hVICs cells after overexpressing hsa_circ_0003748

To determine the effect of hsa_circ_0003748 on the occurrence and development of RVHD, we observed the silencing and overexpression of hsa_circ_0003748 in hVIC cells by synthesizing small interfering RNA (siRNA) and constructing an overexpression plasmid vector pcDNA3.1‐hsa_circ_0003748. After verifying the silencing and overexpression efficiency of hsa_circ_0003748 by qRT‐PCR, we found that expression levels of hsa_circ_0003748 were significantly downregulated and upregulated, respectively (Figure 1A, B). We also designed specific convergent primers for hsa_circ_0003748. Based on hsa_circ_0003748 being encoded from the chromosome 3p21.31 region, the typical transcript is inositol hexakisphosphate kinase 2 (IP6K2) mRNA consisting of 14 exons. hsa_circ_0003748 consists of exons 4 and 5 (Figure S1A). We verified the correctness of RT‐qPCR primers in two ways. First, only one peak was generated based on the melting curve (Figure S1B), suggesting the absence of non‐specific amplification and primer‐dimer formation. The RT‐qPCR product was then analyzed using Sanger sequencing. We found that the product sequence of hsa_circ_0003748 amplified by RT‐qPCR contained a circularization site, and the product sequence was consistent with the original sequence of hsa_circ_0003748 found at the circBase website (http://circr na.org/). These findings indicated that we had designed specific RT‐qPCR amplification primers for hsa_circ_0003748 across the circularization site (Figure S1C).

After silencing and overexpressing hsa_circ_0003748 expression levels, the effect of hsa_circ_0003748 on the progression of RVHD was observed using a CCK‐8 assay. Cell proliferation ability decreased after silencing hsa_circ_0003748 compared with the NC (Figure 1C). After overexpressing hsa_circ_0003748, cell proliferation ability increased compared with the NC (pcDNA3.1) (Figure 1D). This finding suggests that hsa_circ_0003748 might promote RVHD progression.

### 3.2 Hsa_circ_0003748 promotes migration of RVHD cells

**FIGURE 2:**
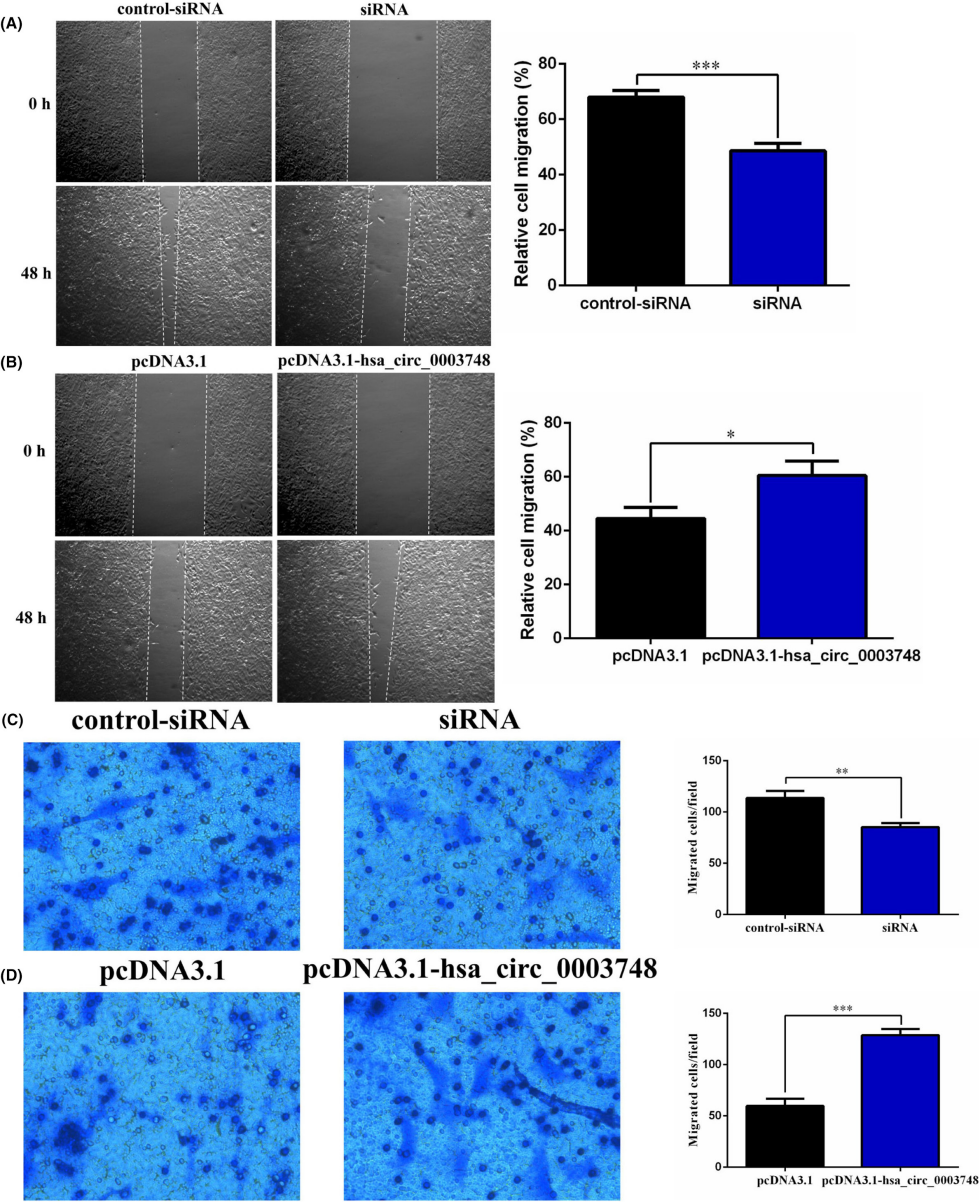
Hsa_circ_0003748 promotes cell migration in hVICs. (A) Changes in relative migration after silencing hsa_circ_0003748 in a wound‐healing assay. (B) Changes in relative migration after overexpressing hsa_circ_0003748 in a wound‐healing assay. (C) Changes in the number of cells migrating into the chamber after silencing hsa_circ_0003748 in a Transwell assay. (D) Changes in the number of cells migrating into the chamber after overexpressing hsa_circ_0003748 in a Transwell assay

We further examined the effect of hsa_circ_0003748 on the migration ability of hVICs. The wound‐healing and Transwell assays showed that, compared with the NC group, migration was reduced after silencing hsa_circ_0003748 (Figure 2A and C). Overexpressing hsa_circ_0003748 increased the migration ability of RVHD cells (Figure 2B and D).

### 3.3 Hsa_circ_0003748 inhibits apoptosis of RVHD cells

**FIGURE 3:**
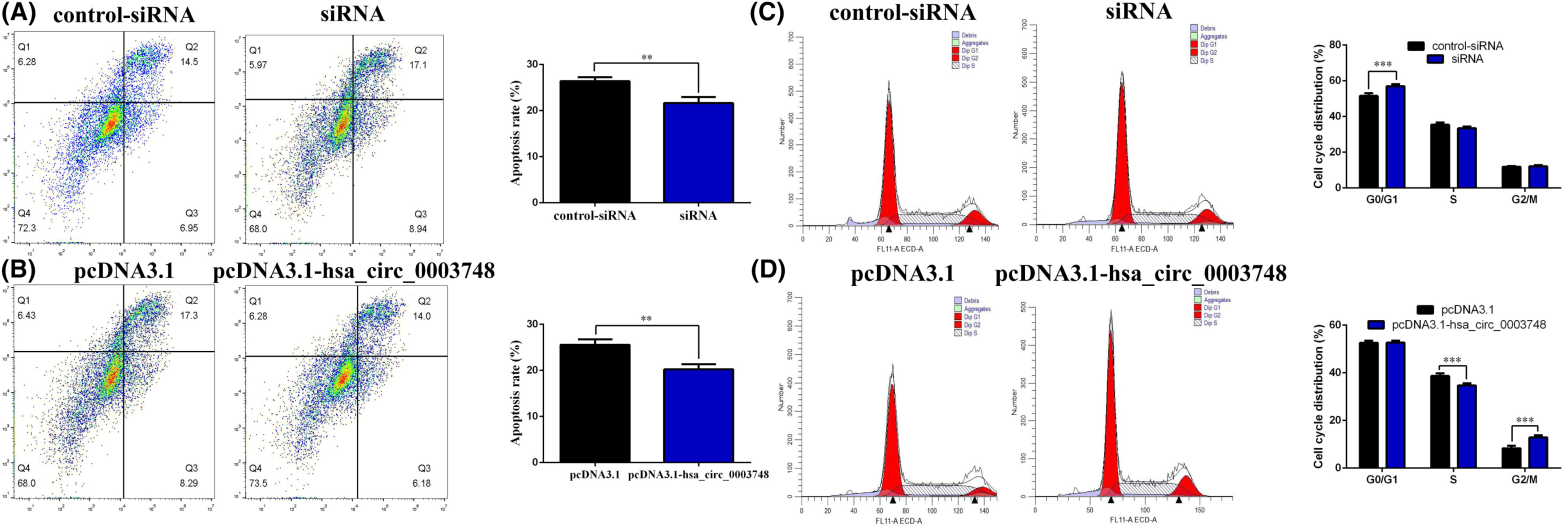
Hsa_circ_0003748 inhibits apoptosis and promotes cycle progression in hVICs. (A) Changes in apoptosis in hVICs cells after silencing hsa_circ_0003748. (B) Changes in apoptosis in hVICs cells after overexpressing hsa_circ_0003748. (C) Changes in the cycle distribution of hVICs cells after silencing hsa_circ_0003748. (D) Changes in the cycle distribution of hVICs cells after overexpressing hsa_circ_0003748

Apoptosis also affects cell proliferation and disease progression. We further detected the effect of hsa_circ_0003748 on the apoptosis of hVIC cells by flow cytometry. We found that the sum of early and late apoptosis rates of hVIC cells increased after silencing hsa_circ_0003748 (Figure 3A); conversely, the sum of early and late apoptosis rates in hVIC cells decreased after overexpression of hsa_circ_0003748 (Figure 3B). These findings suggest that hsa_circ_0003748 inhibits apoptosis in hVICs, promoting RVHD progression.

### 3.4 Hsa_circ_0003748 affects the cycle progression in RVHD cells

Cell cycle distribution determines and influences cell proliferation, which affects disease progression. Using flow cytometry, we found that after silencing hsa_circ_0003748, the cell cycle of hVICs was arrested in the G0/G1 phase (Figure 3C); overexpressing hsa_circ_0003748 arrested the cell cycle in the G2/M phase (Figure 3D). This finding suggests that hsa_circ_0003748 promotes RVHD progression in hVICs.

### 3.5 Hsa_circ_0003748 promotes RVHD progression by sponging miR‐577

**FIGURE 4:**
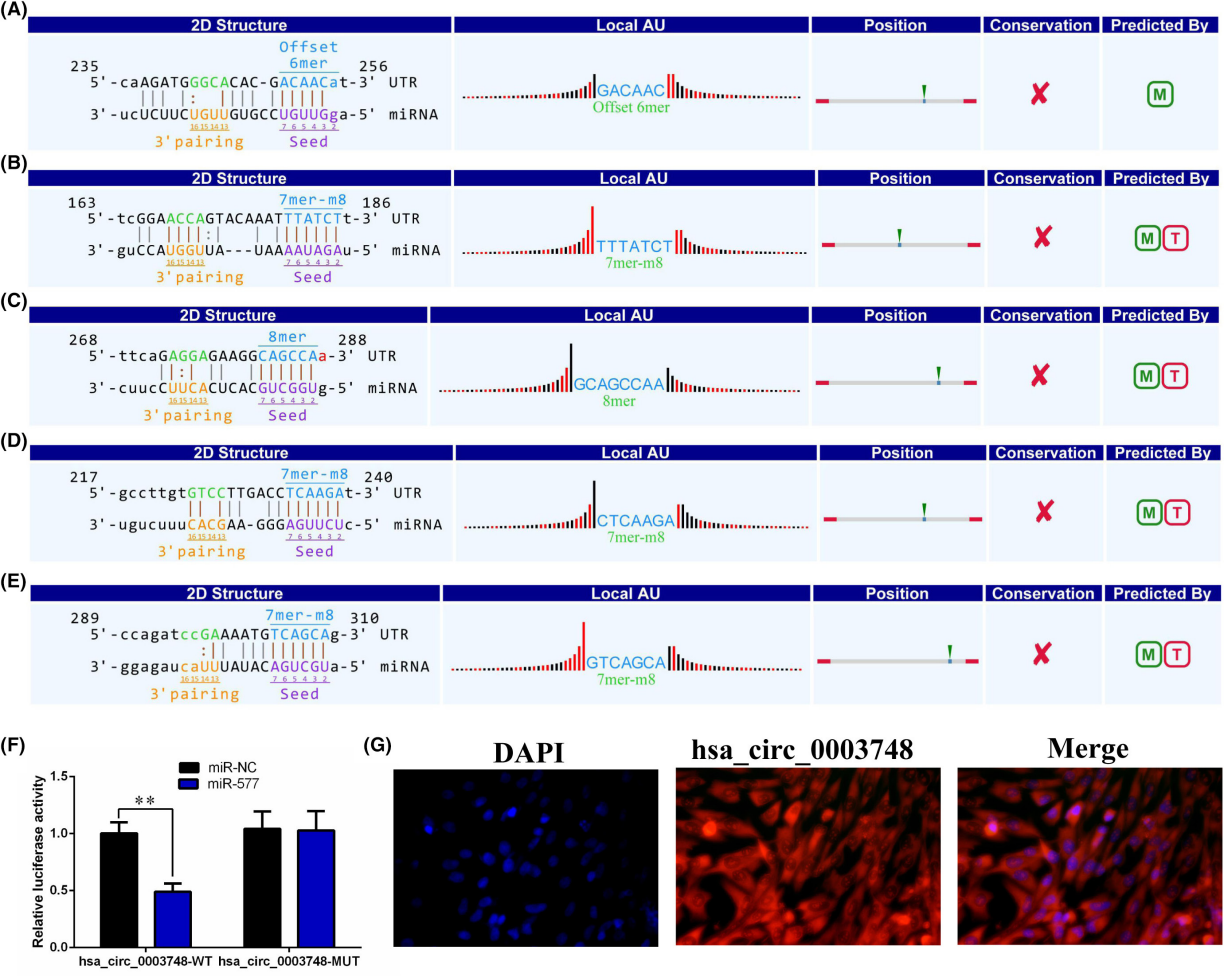
miRNA sponge absorption by hsa_circ_0003748. (A‐E) Bioinformatics prediction of binding of hsa_circ_0003748 and miR‐494‐5p (A), miR‐577 (B), miR‐647 (C), miR‐526b‐5p (D), miR‐628‐5p (E). (F) The dual‐luciferase reporter gene assay confirmed that miR‐577 binds to hsa_circ_0003748. **, *p* < 0.01. (G) FISH experiments confirmed that hsa_circ_0003748 was localized in the cytoplasm. Nuclei were labeled with DAPI and hsa_circ_0006423 with Cy3. The experiments were repeated three times

Using bioinformatics software Circbank and circular RNA interactions, we predicted miRNAs that may act as sponges with hsa_circ_0003748. We obtained five candidate miRNAs: miR‐494‐5p, miR‐577, miR‐647, miR‐526b‐5p, and miR‐628‐5p. We then predicted their possible potential binding sites (Figure 4A‐E). Dual‐luciferase reporter gene experiments showed that miR‐577 and hsa_circ_0003748 have mutual binding sites (Figure 4F). We also verified the localization of hsa_circ_0003748 in cells using a FISH assay and found that hsa_circ_0003748 was present in the cytoplasm (Figure 4G). This finding indicates that hsa_circ_0003748 and miR‐577 may co‐localize in the cytoplasm, and hsa_circ_0003748 promotes RVHD progression by sponging miR‐577.

**FIGURE 5:**
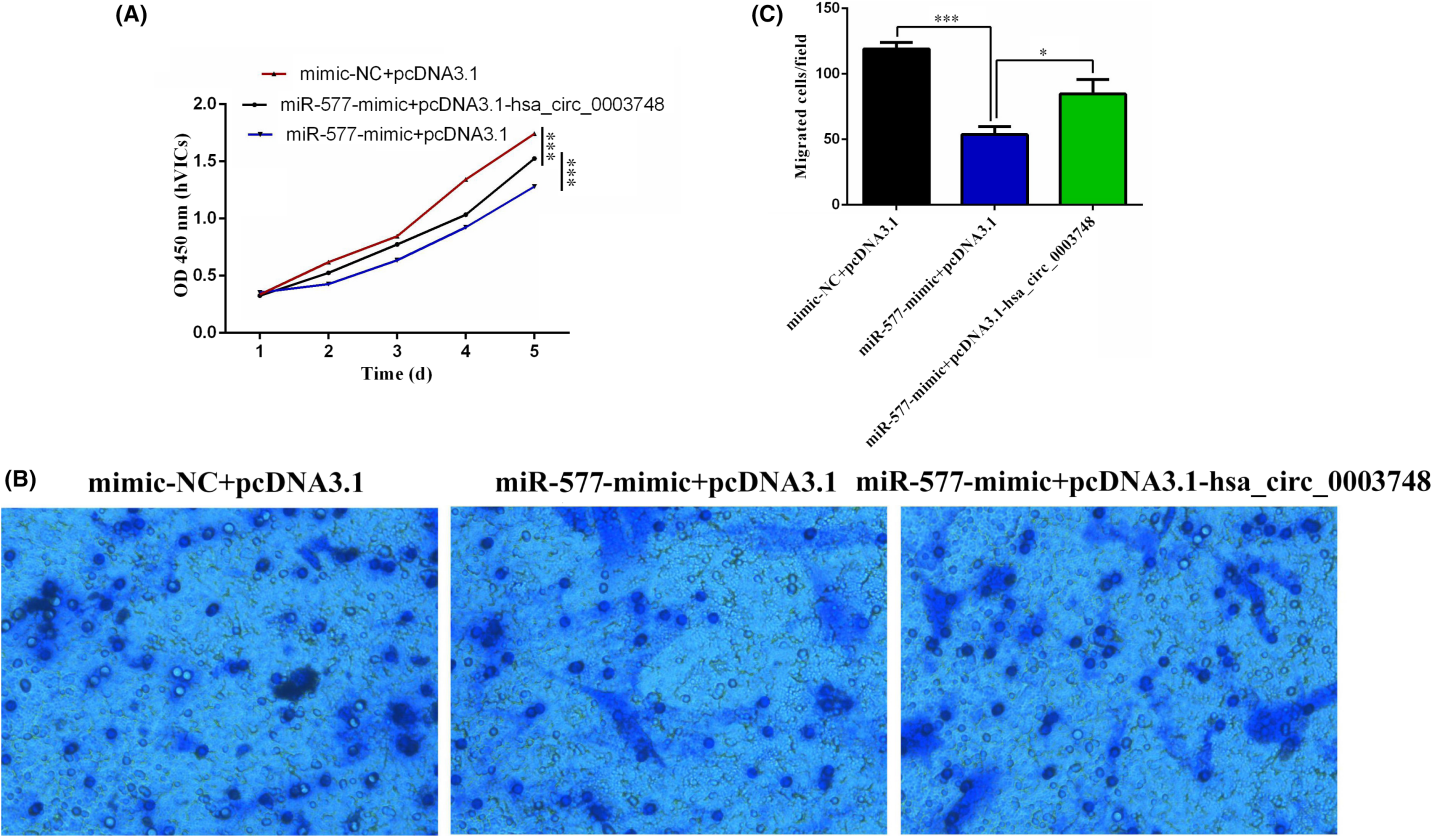
Hsa_circ_0003748 promotes the proliferation and migration ability of RVHD cells by sponging miR‐577. (A) The rescue effect of hsa_circ_0003748 overexpression on miR‐577‐mediated cell proliferation inhibition in hVIC cells was explored using a CCK‐8 assay. (B) The rescue effect of hsa_circ_0003748 overexpression on miR‐577‐mediated cell migration inhibition in hVIC cells was explored using a Transwell assay. (C) Statistical analysis of Transwell assay rescue experiments

To determine whether hsa_circ_0003748 regulates functions after sponging miR‐577, we measured the effects of hsa_circ_0003748 and miR‐577 on proliferation and migration of hVICs cells using rescue experiments. A CCK‐8 assay showed that miR‐577 significantly inhibited the proliferation of hVICs, and this effect was significantly abolished after hsa_circ_0003748 overexpression (Figure 5A). A Transwell assay showed that the inhibition of hVIC cell migration induced by miR‐577 overexpression was alleviated by restoring the expression of hsa_circ_0003748 (Figure 5B, C). These findings suggest that hsa_circ_0003748 promotes proliferation and migration by regulating miR‐577 in RVHD cells.

### 3.6 GO/KEGG enrichment analysis

**FIGURE 6:**
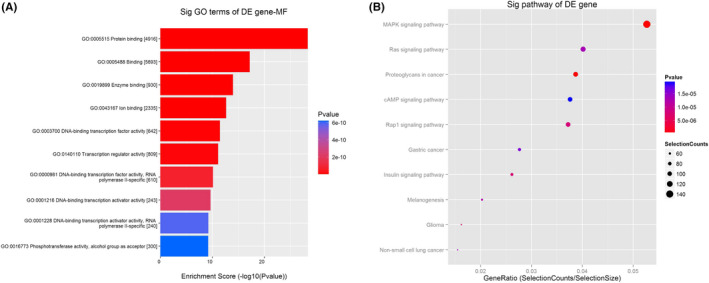
GO and KEGG analyses of relevant biological functions and signaling pathways after hsa_circ_0003748 sponging of miR‐577. (A) GO analysis of biological functions related to hsa_circ_0003748. (B) KEGG analysis of the signaling pathway related to hsa_circ_0003748

We speculated that hsa_circ_0003748 might be involved in biological processes in the progression of RVHD. GO analysis found that these processes include protein binding, enzyme binding, ion binding, DNA‐binding transcription factor activity, and transcription regulator activity (Figure 6A). KEGG analysis revealed that hsa_circ_0003748 might be involved in some signaling pathways in the progression of RVHD. These processes include the MAPK signaling pathway, the Ras signaling pathway, the cAMP signaling pathway, and the Rap1 signaling pathway (Figure 6B).

## 4 DISCUSSION

Rheumatic valvular heart disease is an allergic reaction triggered by group A beta‐hemolytic *Streptococcus* infection. Studies found that the incidence of RVHD varies among people exposed to the same environment and the same lifestyle, suggesting that genetic factors play a role in RVHD.^18^, ^19^ Zhu et al. found that has_circ_0000437 was highly expressed in RVHD and had excellent diagnostic value. In vitro experiments showed that hsa_circ_0000437 promoted proliferation and migration but inhibited apoptosis in RVHD cells.^20^ There is abnormal expression of circRNAs in patients with valvular heart disease complicated with atrial fibrillation. Investigators studied expression profiles of circRNAs, laying the foundation for future circRNA‐related research.^21^ Wang et al. found that melatonin modulated the circRIC3/miR‐204‐5p/DPP4 axis in hVICs to reduce aortic valve calcification, providing a strategy for drug development.^22^

In the present study, 9293 circRNAs were significantly dysregulated in RVHD by obtaining the circRNA microarray detection results from the GEO database. Of these, 4048 were upregulated and 5245 were downregulated. We identified differentially expressed circRNAs with fold‐change >1.2, *p* < 0.05 as the standard and found that 207 were significantly upregulated and 36 were significantly downregulated. Based on circRNA microarray screening, we identified the upregulated circRNA hsa_circ_0003748 in RVHD as the research object. Most circRNAs are expressed by protein‐coding genes and consist of exons.^23^, ^24^ It was determined that hsa_circ_0003748 is an exon‐derived circRNA derived from exon 4 and exon 5 of the IP6K2 gene located on chromosome 3: 48726970–48728915.

In vitro experiments showed that hsa_circ_0003748 promoted the proliferation, migration, and cycle progression of hVICs but inhibited apoptosis. We predicted the existence of binding sites for hsa_circ_0003748 and miR‐577 using bioinformatics software and verified these phenomena using dual‐luciferase reporter gene experiments. We performed GO and KEGG enrichment analysis on the target genes of hsa_circ_0003748 sponge miR‐577 and then analyzed the possible mechanism of hsa_circ_0003748 affecting RVHD progression through sponge miR‐577 from the other side. We found that protein binding, enzyme binding, ion binding, DNA‐binding transcription factor activity, transcription regulator activity, and other biological processes may be involved after hsa_circ_0003748 sponges miR‐577. Therefore, we speculate that hsa_circ_0003748 may affect transcription and the progression of RVHD after sponging miR‐577. Through KEGG analysis, we found that several signaling pathways may be affected by hsa_circ_0003748 sponging miR‐577. Studies found that CD4+ T lymphocytes and the TGF‐β1/MAPK signaling pathway are involved in valve tissue hyperplasia and fibrotic lesions in rheumatic heart disease.^25^ The Ras family of small guanosine triphosphate‐binding proteins in the Ras signaling pathway is essential for intracellular signal transduction for cardiac growth and is involved in the development of cardiac hypertrophy and heart failure. Genetic changes in Ras isoforms or genes in the Ras‐MAPK pathway also lead to heart disease.^26^, ^27^ Lin et al.^28^ found that silencing miR‐665 restored cardiac function in rats with heart failure through the cAMP signaling pathway and overexpression of GLP1R. Qin et al.^29^ found that overexpressing miR‐22‐5p altered the miR‐22‐5p target gene Rap1a and the RAP1/ERK signaling pathway and affected the progression of myocardial injury. Accordingly, we speculated that hsa_circ_0003748 might affect RVHD progression through these pathways by sponging miR‐577.

We believe the hsa_circ_0003748/miR‐577 axis provides a theoretical basis for the genetic diagnosis and treatment of RVHD. In addition, after the hsa_circ_0003748/miR‐577 axis provides a theoretical basis for the genetic diagnosis and treatment of RVHD, we need to further validate our findings with small and large animal experiments. Finally, the feasibility of clinical application can be further verified by clinical trials under ethical circumstances. There remains a lack of research regarding clinical sample verification and in‐depth mechanism; we plan to pursue these goals in future studies.

## CONFLICT OF INTEREST

The authors declare that there is no conflict of interest.

## Supporting information

Fig S1

## Data Availability

The data sets analyzed during the current study are available from the corresponding author upon reasonable request.
