# Supplementary material for: Hsa_circ_0003748 promotes disease progression in rheumatic valvular heart disease by sponging miR‐577
Source: J Clin Lab Anal. 2022 May 9;36(6):e24487. doi: 10.1002/jcla.24487 (PMC9169177; doi:10.1002/jcla.24487)

**Hsa_circ_0003748 promotes disease progression in rheumatic valvular heart disease by sponging miR-577**

**Supplementary Information**

Supplementary Figure 1. Amplification of hsa_circ_0003748. (A) Hsa_circ_0003748 and convergent primers. hsa_circ_0003748-derived transcript, the forward primer spans the splice site sequence. (B) Melting curve of hsa_circ_0003728 RT-qPCR, a total of three representative samples. (C) Sanger sequencing result of hsa_circ_0000437 RT-qPCR product; arrows represent circularization sites.


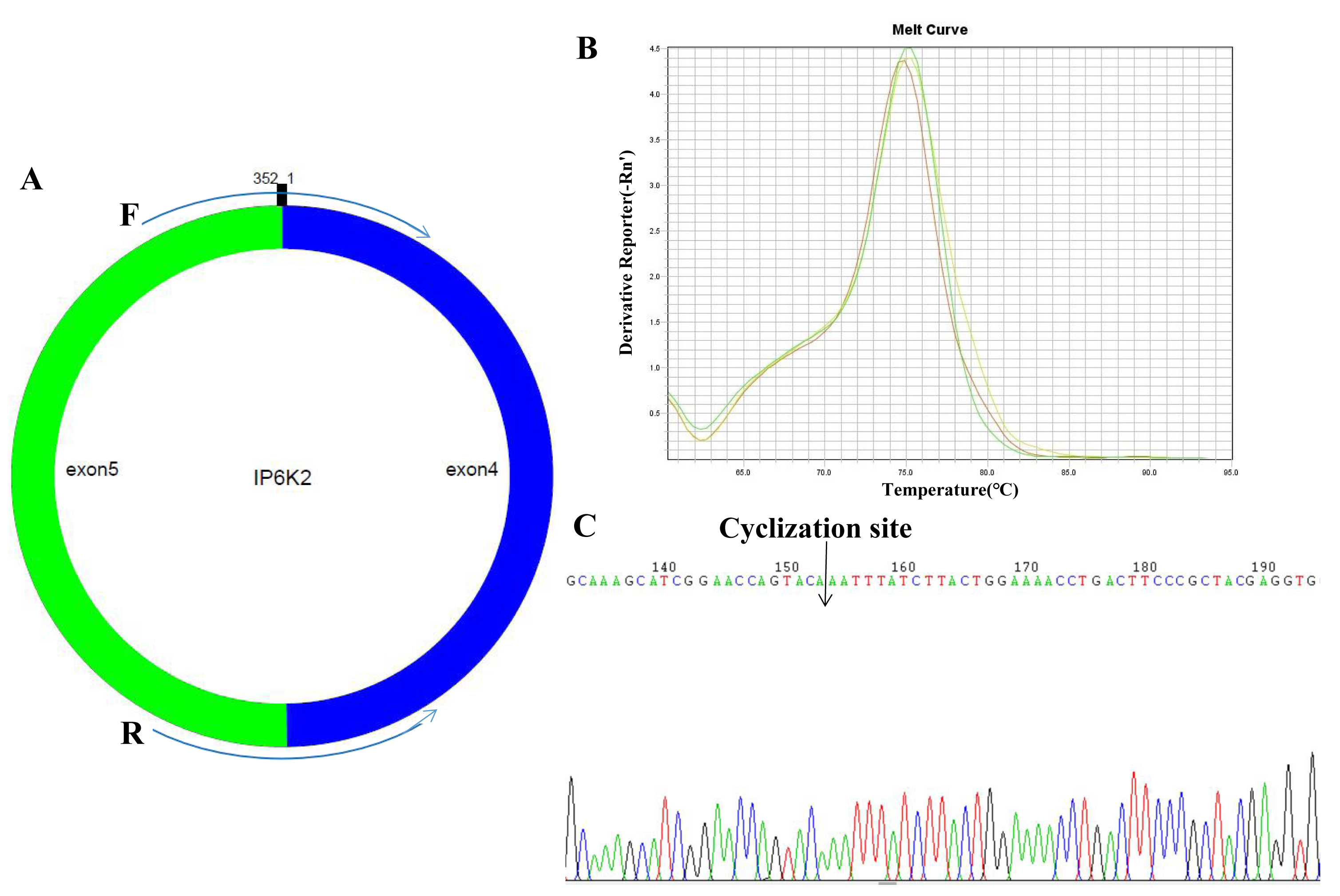

Supplement: Supplementary file 1 — Fig S1 [file JCLA-36-e24487-s001.doc]
